# Der außergewöhnliche Nachahmer einer Polymyositis

**DOI:** 10.1007/s00393-022-01173-w

**Published:** 2022-03-29

**Authors:** S. T. Jendrek, M. Glatzel, D. Pauli, S. Schinke, J. Y. Humrich, P. Lamprecht, G. Riemekasten

**Affiliations:** 1 Klinik für Rheumatologie und klinische Immunologie, Universitätsklinikum Schleswig-Holstein, UKSH, Campus Lübeck, Ratzeburger Allee 160, 23562 Lübeck, Deutschland; 2 Zentrum für Diagnostik, Institut für Neuropathologie, Universitätsklinikum Hamburg-Eppendorf, Hamburg, Deutschland; 3 Institut für Klinische Chemie (Zentrallabor), Universitätsklinikum Schleswig-Holstein, Campus Lübeck, Lübeck, Deutschland

**Keywords:** Hantavirus, Myalgien, Myositis, Diagnose, Immunsuppression, Hantavirus, Myalgias, Myositis, Diagnosis, Immunosuppression

## Abstract

Beschrieben wird eine Hantavirus-assoziierte, ausgeprägte Myositis als seltene Differenzialdiagnose zu einer Polymyositis. In der Literatur wird für die Pathogenese der Hantaviruserkrankung weniger eine direkte virale Zytopathologie als eine sekundäre Immundysregulation mit Induktion eines Kapillarlecks diskutiert. Wir beschreiben mit diesem Fall erstmalig die erfolgreiche Behandlung einer protrahiert verlaufenen Hantavirus-Myositis mittels Einsatzes von hoch dosierten Glukokortikoiden und Cyclophosphamid, gefolgt von Ciclosporin und Methotrexat (MTX).

## Abstract (en)

This article describes a hantavirus-associated pronounced myositis as a rare differential diagnosis to polymyositis. The literature on the pathogenesis of hantavirus disease discusses less a direct viral cytopathology but more a secondary immune dysregulation with induction of a capillary leak. This article describes for the first time a case of successful treatment of protracted hantavirus myositis using high-dose glucocorticoids and cyclophosphamide, followed by ciclosporin and MTX.

## Falldarstellung

### Anamnese – Krankheitsverlauf

Ein 56-jähriger Patient stellte sich Ende Dezember 2017 mit seit knapp 3 Wochen zunehmenden, stammnahen Myalgien und Muskelschwäche der Oberarme und Oberschenkel sowie der Hüftmuskulatur vor. Begleitend bestanden eine schwere Abgeschlagenheit und subfebrile Temperaturen. Die Symptome hatten plötzlich mit hohem Fieber und generalisierten Gliederschmerzen sowie passageren Halsschmerzen eingesetzt. Innerhalb von 3 Tagen besserten sich die Beschwerden bis auf die proximalen Myalgien, welche weiter zunahmen. Das Integument war unauffällig. Vorerkrankungen waren keine bekannt. Medikamenteneinnahmen oder Alkoholkonsum bestanden nicht. Auslandsaufenthalte wurden neben besonderen Freizeitaktivitäten und Sport verneint. Der Patient war selbstständiger Landwirt.

### Initiale Befunde

Pathologische Laborwerte waren: eine Thrombo- und Lymphopenie (113/nl bzw. 0,54/nl), eine CK-Erhöhung (4022 U/l, >20-fach über der Norm), eine Erhöhung von Myoglobin (538 µg/l) sowie der Transaminasen (GPT 160 U/l, GOT 294 U/l). Normwertig waren: Serumbilirubin, C3/4, D‑Dimere, Troponin T, NTproBNP, Gamma-GT, AP, Nierenretentionsparameter sowie C‑reaktives Protein. Unter der Arbeitsdiagnose einer Myositis erfolgte zunächst neben einer infektiologischen Differenzialdiagnostik (Adeno‑/Enteroviren, Coxsackievirus, EBV, CMV, Parvovirus B19, Influenza A + B, Parainfluenza, Hepatitis B/C/E, HIV, *Treponema pallidum*, Leptospiren, Borrelien) auch die Bestimmung der ANA/ENA und des Myositisprofils. Zusammenfassend waren hierbei keine pathologischen Befunde zu erheben.

### Verlauf, Diagnose und Therapie

**Figure 1:**
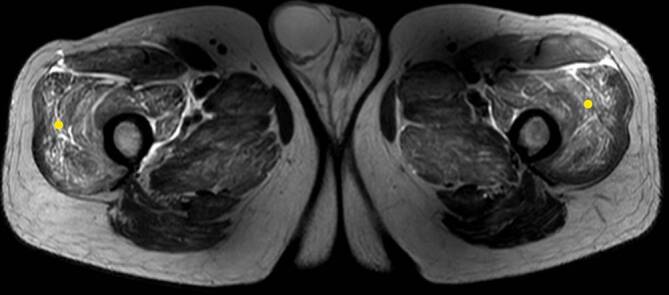

Nachdem der vormals gesunde Patient durch die ausgeprägten Myalgien unter einer zunehmenden Immobilisation bis zur Bettlägerigkeit litt und die CK auf unverändert hohem Niveau stagnierte, entschieden wir uns zu einer hoch dosierten Glukokortikoid-Stoßtherapie (initial 250 mg Methylprednisolon täglich i.v. für 4 Tage). Die Muskel-MRT der Becken- und Beinregion sowie eine EMG-Untersuchung erbrachten das Bild einer ausgeprägten, symmetrischen Myositis (Abb. 1) und eines myopathisch veränderten Potenzials der motorischen Einheit (PmE). Das ergänzte Myositis-Autoantikörperprofil war ebenso wie die ANCA, Kryoglobuline und HMG-CoA-Reduktase-Antikörper negativ. In der Lungenfunktion und pulmonalen Bildgebung zeigten sich keine Auffälligkeiten im Sinne einer komplizierenden Organbeteiligung einer Myositis. Eine Muskelbiopsie und dezidierte Tumorsuche lehnte der Patient zunächst ab.

Am 3. Tag nach Einleitung der Glukokortikoid-Stoßtherapie kam es zu einem signifikanten Abfall der CK auf 1093 U/l. Der Patient konnte schließlich in deutlich gebessertem Allgemeinzustand und bei nahezu sistierenden Myalgien (Ck 727 U/l) mit einer Prednisolon-Dosis von 50 mg/Tag unter Mitgabe eines Prednisolon-Reduktionsschemas entlassen werden. Da der Patient als Landwirt einer beruflichen Risikogruppe angehörte, wurde eine Hantavirus-Serologie ergänzt. Überraschenderweise bestätigte sich serologisch eine kürzlich zurückliegende Hantavirus-Infektion (Hantavirus Blot IgM/IgG positiv; Immunoblot recomLine HantaPlus IgG und IgM (Mikrogen, Neuried, Deutschland)).

Im Immunoblot wurde eine IgG- und IgM-Positivität für die Serotypen Dobrava, Hantaan und Sin Nombre nachgewiesen. Die PCR auf Hantavirus-RNA im Blut fiel negativ aus. Ein typisches hämorrhagisches Fieber mit renalem Syndrom (HFRS) oder ein Hantavirus-assoziiertes pulmonales Syndrom (HPS) lag nicht vor. Im Verlauf kam es zu einem Verlust des Hantavirus-IgM, sodass auch bei negativer PCR (= häufiger Befund bei stets nur sehr kurzer Virämie [1]) von einer frischen Infektion auszugehen war.

**Figure 2:**
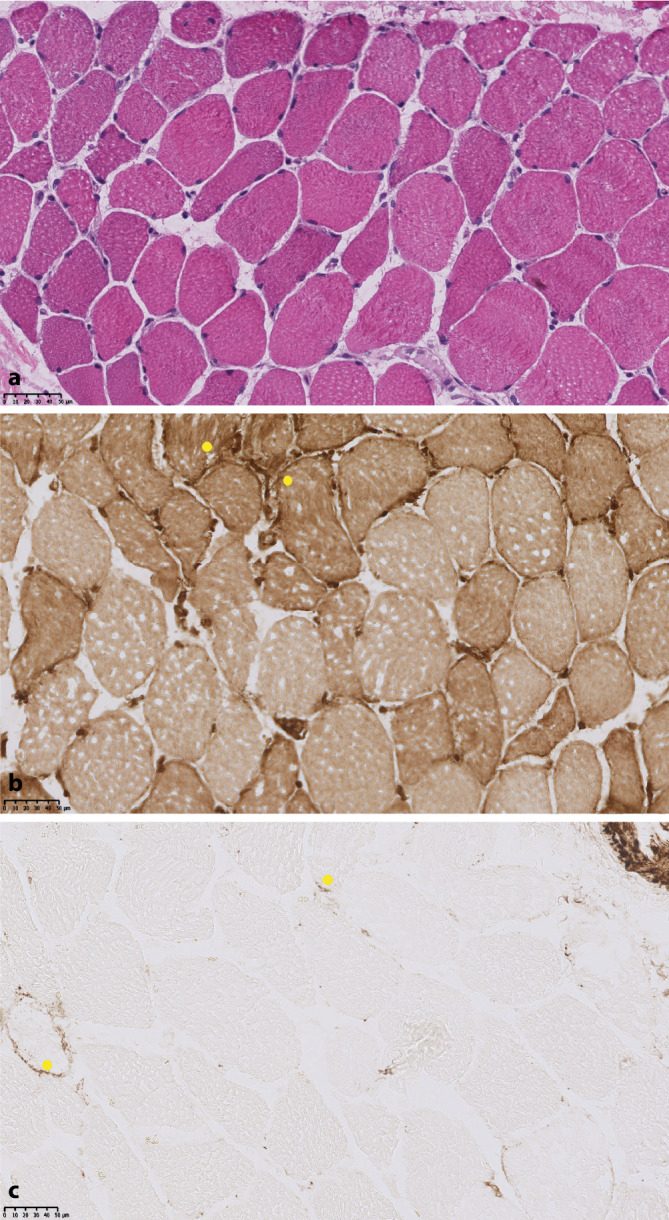

Zwei Wochen später erfolgte eine Wiederaufnahme mit stärkeren Myalgien der vorbeschriebenen Lokalisationen, zunehmender Kraftlosigkeit und Palpitationen. Die Prednisolon-Dosis zu diesem Zeitpunkt betrug noch 30 mg/Tag. Laborchemisch war die CK erneut auf 1364 U/l gestiegen. Darüber hinaus fiel erstmalig eine Erhöhung des Troponin T auf das 16-Fache der Norm (225 ng/l) auf. Elektrokardiographisch (inklusive LZ-EKG) und echokardiographisch lagen Normalbefunde vor. Eine Kardio-MRT wurde aufgrund von Platzangst abgelehnt. Der Patient stimmte nun einer Muskelbiopsie zu, welche den Befund einer floriden, a.e. autoimmunen Myopathie erbrachte (Abb. 2 und 3). Die PCR auf Hantavirus-RNA aus den Muskelbiopsat sowie erneut aus dem Blut fiel negativ aus. Wir wiederholten eine 4‑tägige intravenöse Methylprednisolon-Stoßtherapie à 250 mg mit begleitender Gabe von intravenösen Immunglobulinen (IVIG). Bei refraktärer Myositis ohne Anhalt für eine Viruspersistenz mit nun neu hinzugetretener vital bedrohlicher kardialer Beteiligung folgte zur Remissionsinduktion eine intensivierte Immunsuppression mittels Einsatzes von Ciclosporin (50 mg 2‑mal täglich p.o.) in Kombination mit Cyclophosphamid (Bolustherapie mit 1000 mg i.v. in 2‑ bis 3‑wöchigem Intervall nach CYCLOPS-Schema). Insgesamt erfolgten 6 Gaben Cyclophosphamid à 1000 mg unter fortgesetzter Ciclosporin-Therapie und langsamer Prednisolon-Reduktion. Einer raschen CK-Normalisierung bereits nach der 3. Cyclophosphamid-Gabe folgte eine Normalisierung des Troponin T nach 12 Wochen unter fortgesetzter Prednisolon-Reduktion. Nach Erreichen einer Remission und Beendigung der Cyclophosphamid-Therapie (Kumulativdosis = 6 g) setzten wir bei einer täglichen Prednisolon-Einnahme von 5 mg/Tag nach 17 Wochen eine Remissionserhaltung mit Ciclosporin (50 mg 2‑mal täglich p.o.) und MTX (20 mg s.c. pro Woche) fort. Nach Ausschleichen des Prednisolon wurde nach 9 Monaten die Therapie mit MTX und bei stabiler Remission nach weiteren 3 Monaten Ciclosporin komplikationslos beendet. Regelmäßige klinische/laborchemische Kontrollen erbrachten bis zuletzt keinen Anhalt für eine neuerliche Myositisaktivität.

**Figure 3:**
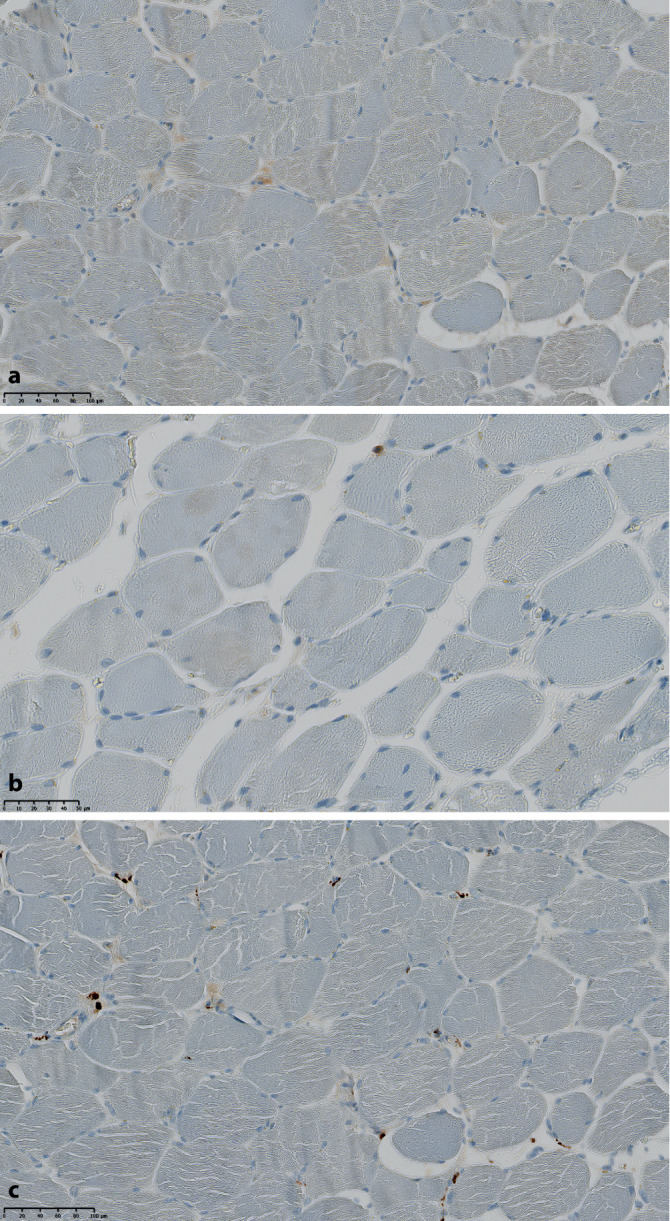

## Diskussion/Zusammenfassung

Das Auftreten einer derart ausgeprägten Hantavirus-assoziierten Myositis wurde bislang nur selten beschrieben [4]. Bei betroffenen Patienten war zudem ein HPS mit der Notwendigkeit einer invasiven Beatmung vordergründig. Supportiv erfolgten nur parenterale Volumen- und Antibiotikagaben. Mit Besserung des HPS war bei diesen Patienten zwar ein Rückgang der CK beschrieben, wobei jedoch eine weitergehende Myositisdiagnostik und Verlaufskontrollen der CK nicht erfolgt waren.

Mit unserem Fall beschreiben wir nun erstmals die erfolgreiche Behandlung einer protrahierten Hantavirus-assoziierten Myositis mit kardialer Beteiligung mittels Cyclophosphamid und Ciclosporin mit flankierender Glukokortikoid- und IVIG-Therapie. Die Hantaviruserkrankung hat sich im vorliegenden Fall untypisch manifestiert. Die berufliche Risikotätigkeit (Landwirt mit Kontakt zu Nagetierausscheidungen) veranlasste uns jedoch zur Durchführung einer entsprechenden Serologie. Hinsichtlich der Pathogenität des Hantavirus ist die Auslösung eines Kapillarlecksyndroms bedeutsam [2]. Ferner existieren Hinweise darauf, dass insbesondere die T‑Zell-Aktivität [6] und Zytokine wie der Tumornekrosefaktor‑α (TNF-α), Interleukin(IL)-6, IL-10 und Interferon‑γ eine wichtige Rolle in der Pathogenese der Hantaviruserkrankung spielen [5]. In Deutschland sind v. a. Infektionen mit den Serotypen Puumala-Virus und einer Form des Dobrava-Belgrad-Virus vorherrschend [3].

In unserem Fall war von einer Dobrava-Infektion auszugehen, da ein Aufenthalt außerhalb Europas ausgeschlossen werden konnte. Der Immunoblothersteller gibt in diesem Fall an, dass andere Serotypen als die in Deutschland endemischen Varianten als Kreuzreaktivität zu bewerten sind.

Serologisch wiesen auf die kürzlich abgelaufene akute Hantavirus-Infektion rückläufige IgM-Antikörper in einer Kontrolle 2 Monate nach Erstvorstellung hin. Der ausbleibende direkte Erregernachweis mittels PCR war hingegen nicht ungewöhnlich, da die Hanta-Virämie stets nur kurzfristig nachzuweisen ist.

## Fazit für die Praxis

- Eine Hantavirus-assoziierte Myositis kann klinisch und apparativ das Bild einer Polymyositis imitieren, sodass bei unklaren Myositiden und ggf. Risikokonstellation (z. B. Berufsexposition) an die Durchführung einer Hantaviren-Serologie gedacht werden sollte.
- Im vorliegenden Fall war eine Remissionsinduktion der Hantavirus-assoziierten Myositis mit kardialer Beteiligung mittels Methylprednisolon, Ciclosporin und Cyclophosphamid effektiv und gut verträglich.

## Abkürzungen

AP: Alkalische Phosphatase
Ck: Kreatinkinase
CMV: Zytomegalievirus
EBV: Epstein-Barr-Virus
EMG: Elektromyographie
GOT: Glutamat-Oxalacetat-Transaminase
GPT: Glutamat-Pyruvat-Transaminase
MTX: Methotrexat
